# Differential Modulation of Arcuate Nucleus and Mesolimbic Gene Expression Levels by Central Leptin in Rats on Short-Term High-Fat High-Sugar Diet

**DOI:** 10.1371/journal.pone.0087729

**Published:** 2014-01-31

**Authors:** José K. van den Heuvel, Leslie Eggels, Eric Fliers, Andries Kalsbeek, Roger A. H. Adan, Susanne E. la Fleur

**Affiliations:** 1 Department of Endocrinology and Metabolism, Academic Medical Center, University of Amsterdam, Amsterdam, The Netherlands; 2 Hypothalamic Integration Mechanisms, Netherlands Institute for Neuroscience, Amsterdam, The Netherlands; 3 Rudolf Magnus Institute of Neuroscience, Department of Neuroscience and Pharmacology, University Medical Centre Utrecht, Utrecht, The Netherlands; Monash University, Australia

## Abstract

**Objective:**

Leptin resistance is a common hallmark of obesity. Rats on a free-choice high-fat high-sugar (fcHFHS) diet are resistant to peripherally administered leptin. The aim of this study was to investigate feeding responses to central leptin as well as the associated changes in mRNA levels in hypothalamic and mesolimbic brain areas.

**Design and Methods:**

Rats on a CHOW or fcHFHS diet for 8 days received leptin or vehicle intracerebro(lateral)ventricularly (ICV) and food intake was measured 5 h and 24 h later. Four days later, rats were sacrificed after ICV leptin or vehicle and mRNA levels were quantified for hypothalamic pro-opiomelanocortin (POMC) and neuropeptide Y (NPY) and for preproenkephalin (ppENK) in nucleus accumbens and tyrosine hydroxylase (TH) in ventral tegmental area (VTA).

**Results:**

ICV leptin decreased caloric intake both in CHOW and fcHFHS rats. In fcHFHS, leptin preferentially decreased chow and fat intake. Leptin increased POMC and decreased NPY mRNA in CHOW, but not in fcHFHS rats. In CHOW rats, leptin had no effect on ppENK mRNA and decreased TH mRNA. In fcHFHS, leptin decreased ppENK mRNA and increased TH mRNA.

**Conclusion:**

Despite peripheral and arcuate leptin resistance, central leptin suppresses feeding in fcHFHS rats. As the VTA and nucleus accumbens are still responsive to leptin, these brain areas may therefore, at least partly, account for the leptin-induced feeding suppression in rats on a fcHFHS diet.

## Introduction

Since the discovery of leptin in 1994 [1] a wealth of studies has been generated on leptin’s role in feeding behavior and metabolism. Leptin is secreted from adipose tissue and exerts several metabolic functions in the body, including the regulation of satiety via the brain. Although initially considered as a potential anti-obesity drug, the development of leptin resistance in obesity has limited this perspective so far. Leptin resistance is the process occurring in human obesity and in several models of rodent obesity in which synthesis and secretion of leptin are increased, while inhibition of feeding or activation of energy expenditure by leptin are decreased [2], [3]. The underlying mechanisms of this resistance have not been completely elucidated yet.

Leptin’s actions in the brain have been best characterized in the arcuate nucleus of the hypothalamus, where leptin suppresses feeding by acting on leptin receptors on neuropeptide Y (NPY)/Agouti related protein (AgRP) neurons and pro-opiomelanocortin (POMC) neurons. Both NPY and AgRP stimulate feeding, whereas alpha-MSH, which is a product of the POMC gene after splicing, suppresses feeding. Leptin exerts its inhibitory effects on feeding by changing the excitability of the POMC and NPY/AgRP neurons and by increasing and decreasing their expression levels, respectively [4].

Although the emphasis for leptin’s action concerning its effect on feeding behavior has mainly been on the hypothalamus and brainstem [5], [6], leptin receptors are more widely spread throughout the central nervous system including the hippocampus and ventral tegmental area (VTA). Within the VTA, leptin receptors are colocalized with tyrosine hydroxylase (TH, a precursor in the synthesis of dopamine) neurons [7]–[9], which project to the nucleus accumbens and are important for food motivated behavior [10]. Leptin modulates dopamine dependent measures of food and drug reward [8] and has been shown to affect VTA dopamine content [11], firing rate [7] and high fat food preferences [12]. Moreover, leptin-induced changes in TH levels have been observed, although the exact nature of these changes remains to be elucidated, as selective up-regulation [8], [13] and downregulation [14] of TH levels have been reported.

Previous research showed that rats offered saturated fat and a sugar solution in addition to a chow diet (a free-choice high-fat high-sugar (fcHFHS) choice diet) for only 8 days are peripherally leptin resistant [3]. Peripheral leptin resistance is defined as a lack of feeding suppression upon peripherally (intraperitoneally) injected leptin [3]. The first aim of this study was to investigate whether central administered leptin in rats on a fcHFHS diet would exert an effect on food intake. Since, we did observe food intake inhibition after ICV leptin in rats on a fcHFHS diet comparable to that for rats on CHOW, we next aimed to determine the response to ICV leptin at the gene level in rats on fcHFHS and CHOW diet, focusing on hypothalamic and mesolimbic brain areas.

As leptin did exert effects when injected centrally but not peripherally ([3] and our own unpublished observations), we first focused on leptin’s central effects on arcuate nucleus NPY and POMC expression. As leptin did not affect neuropeptide expression in the arcuate nucleus in rats on the fcHFHS diet but leptin did specifically inhibit chow intake and fat intake, we next focused on the midbrain dopaminergic system as part of the reward pathway and determined TH mRNA levels in the VTA. Additionally, as leptin has been shown to affect nucleus accumbens dopamine flux [13], opioid neurons are influenced by dopamine and fat intake is linked to nucleus accumbens opioid regulation [15], we determined ppENK (precursor for enkephalin) expression in the nucleus accumbens as well.

## Methods and Procedures

### Animals and Dietary Intervention

Male Wistar rats (Harlan, Horst, the Netherlands) weighing 250–280 g were individually housed in Plexiglas cages in a temperature (21–23°C) and light-controlled room (lights on 0700–1900). Rats were subjected to either a free-choice high-fat high-sugar diet (fcHFHS) diet or a control diet (CHOW), as has previously been described [16]. The experiments were approved by the Committee for Animal Experimentation of the Academic Medical Center of the University of Amsterdam.

### Surgery and Procedure for Intracerebroventricular (ICV) Injections

One week after arrival, rats received a cannula aimed at the lateral ventricle. Rats were anaesthetized with an intraperitoneal (i.p.) injection of 80 mg/kg Ketamin (Eurovet Animal Health, Bladel, the Netherlands), 8 mg/kg Xylazin (Bayer Health Care, Mijdrecht, the Netherlands) and 0.1 mg/kg Atropin (Pharmachemie B.V., Haarlem, the Netherlands), and fixed in a stereotaxic frame. A permanent 22-gauge stainless steel guide cannula (Plastics One, Bilaney Consultants GmbH, Dusseldorf, Germany) was implanted into the right lateral ventricle with coordinates: 0.8 mm posterior from Bregma, 1.4 mm lateral from midline and 5 mm below the surface of the brain. The guide cannula was secured to the skull using three anchor screws and dental cement. A 28-gauge stainless steel dummy cannula, extending 0.5 mm beyond the guide, was used to occlude the guide cannula. Immediately after surgery, rats received an analgesic (Carprofen, 0.5 mg/100 g BW) and were housed individually.

Rat leptin was obtained from Dr. Parlow (NIDDK, http://www.humc.edu./hormones) and was dissolved in phosphate buffered saline (PBS), which also served as the vehicle control solution. All ICV injections were delivered in a volume of 3 µl. The day before each ICV injection at 5 PM, all rats were food restricted O/N and received 10 grams of chow. The next morning, in the beginning of the light phase (between 10 AM and 11 AM), rats received an ICV injection of leptin or vehicle in randomized order. All individual food components were measured after 5 h and 24 h and caloric intake (kcal) for each individual food item and total caloric intake calculated. Cannula placement was validated by inspection of coronal serial sections subjected to thionin staining.

### Effect of Central Leptin Administration on Food Intake and Gene Expression Levels

After performing a dose response with 10 µg and 15 µg leptin one week after the stereotactic operation, rats were randomly divided into two groups (CHOW or fcHFHS) and were maintained on their respective diets throughout the remainder of the experiment. After 7 days on the diet, rats were food restricted O/N (as described above) and the next morning (on day 8), rats received ICV injection of 15 µg leptin or vehicle in randomized order and food intake was measured 5 h and 24 h later. In a separate experiment, the effect of leptin on gene expression levels was determined. To allow the first leptin dose (of day 8) to completely fade out, we waited three days after the first experiment. Subsequently, rats were again food restricted on day 11 and received ICV leptin or vehicle on day 12. Between ICV and decapitation two hours later, food was not returned. Decapitation occurred under CO2/O2 and brains were quickly removed and frozen on dry ice and used for *in situ* hybridization. Individual mesenteric-omental, epididymal, subcutaneous (inguinal) and perirenal white adipose tissues were dissected, cleaned and weighed. Trunk blood was centrifuged and plasma was stored at −20°C. Plasma concentrations of leptin were determined as previously described [17].

### *In situ* Hybridization

Coronal sections (20 µm) were used for radioactive *in situ* hybridization and sections were obtained according to Paxinos and Watson, 4^th^ edition (1998). Sections were defrosted and fixed in 4% paraformaldehyde (PFA) in phosphate-buffered saline (PBS) for 10 min, washed in PBS, pretreated with 0.25% acetic anhydride in 0.1 M triethanolamine, washed again in PBS and dehydrated in graded ethanol followed by 100% chloroform and 100% ethanol. A ^33^P-labeled RNA probe (^33^P-UTP, Perkin Elmer) was made using a 287 bp rat NPY, 350 bp rat POMC, 800 bp rat ppENK or 1200 bp TH cDNA fragment. The sections were hybridized overnight at 72°C with 10^6^ cpm probe in buffer containing 50% deionized formamide, 2× standard saline citrate (SSC), 10% dextrane sulphate, 1× Denhardt's solution, 5 mM EDTA and 10 mM phosphate buffer, after 5 min heating at 80°C. After hybridization, the sections were washed in 5×SSC (short, 72°C) and 0.2×SSC (2 h, 72°C) and dehydrated in graded ethanol in 0.3 M ammonium acetate. Sections were exposed to X-ray film (Kodak Bio-Max MR) for 5 days for POMC and 4 days for NPY, ppENK and TH. The films were developed and expression levels were quantitatively analyzed using an 8800F Canon scanner. All images (600 dpi) were analyzed using ImageJ (Rasband, WS, NIH, Bethesda, MD, USA, http://rsbweb.nih.gov/ij/, 1997–2005). In each section gray values were determined in the region of interest, measured bilaterally and subtracted from background, producing a single value for each brain area on each section.

### Statistical Analysis

Data are presented as mean ± standard error of the mean (SEM). The effect of leptin on caloric intake was analyzed using a two-way repeated-measures analysis of variance (ANOVA) with time (5 hour and 24 hour) as within-animal variable and diet (fcHFHS-CHOW) and drug (Leptin-PBS) as between-animal factors. To determine the effects of diet and leptin on gene expression analysis, a two-way ANOVA was performed. Data were analyzed by one-way ANOVA when single outcome measurements or separate time points between the groups were compared. A significant (P<0.05) global effect of the repeated-measurements or one-way ANOVA was followed by post hoc tests for individual group differences (Fisher's protected least significant difference). Significance was defined at P<0.05.

## Results

### Body Weight and Feeding Behavior

**Figure 1:**
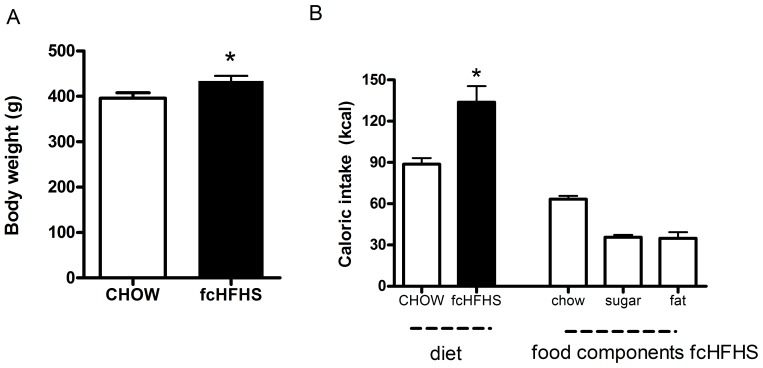
Body weight and caloric intake in rats on CHOW or fcHFHS diet. A) Body weight after 12 days CHOW or fcHFHS diet. B) Averaged daily caloric intake in rats on CHOW and fcHFHS diet and absolute amounts of daily total caloric intake of the individual food components chow, sugar solution and saturated fat in rats on a fcHFHS diet. Data are mean ± SEM.

Rats on the fcHFHS diet were significantly heavier than rats on the CHOW control diet (Table 1, Figure 1a, P<0.05). Average daily total caloric intake in rats on fcHFHS diet was higher than in rats on CHOW diet (Table 1, Figure 1b, P<0.05). There were no significant differences in food intake and body weight between day 8 and day 12.

**Table 1:** Effect of free choice high-fat high-sugar diet (fcHFHS) on body weight and adiposity.

	CHOW	fcHFHS
*n*	15	18
Body weight (g)	396±12	434±11*
Δ Body weight (g)	53±4.4	68±4.8*
% chow intake	100	44.2
% sugar intake		28.6
% fat intake		27.2
Mesenteric fat (g)	2.68±0.14	4.82±0.36*
Mesenteric fat (% of BW)	0.67±0.03	1.06±0.08*
Perirenal fat (g)	2.43±0.17	4.55±0.31*
Perirenal fat (% of BW)	0.57±0.04	0.94±0.06*
Epididymal fat (g)	2.93±0.10	4.55±0.31*
Epididymal fat (% of BW)	0.79±0.04	1.15±0.08*
Subcutaneous fat (g)	2.64±0.14	4.66±0.45*
Subcutaneous fat (% of BW)	0.64±0.02	0.98±0.12*
Total fat (g)	10.70±0.46	19.18±1.38*
Total fat (% of BW)	2.67±0.13	4.13±0.31*
Leptin, ng/ml	2.48±0.53	6.76±1.55*

Values are mean ± SEM.

* P<0.05 compared to CHOW control group.

### Effect of Central Leptin on Food Intake after 8 Days fcHFHS or CHOW Diet

**Figure 2:**
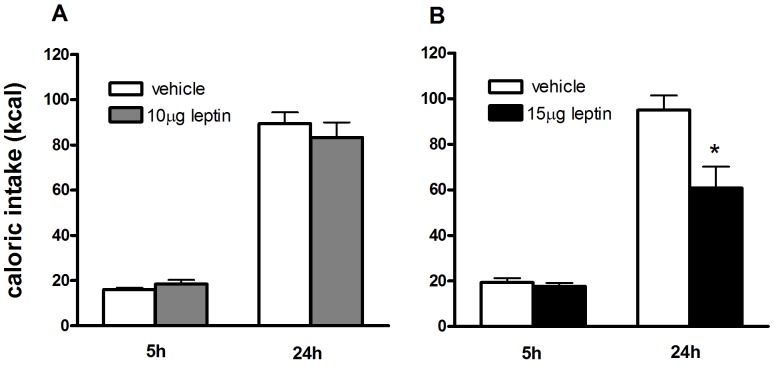
Determination of effective dose of ICV leptin to suppress feeding. A) The dose of 10 µg leptin did not elicit food intake suppression at any of the time points measured. B) 15 µg leptin significantly suppressed food intake 24 hours after administration. Therefore a dose of 15 µg was used in the following experiments. *P<0.05, vehicle vs. leptin. Data are mean ± SEM.

We here show effects of leptin administered in the lateral ventricle, thus providing leptin to all brain areas. We started with testing two leptin doses (10 µg and 15 µg) and showed that a dose of 15 µg leptin significantly decreased cumulative chow intake at t = 24 h (P<0.05)(Figure 2). Therefore, in the following experiments the dose of 15 µg leptin was used. This dose is comparable to studies showing ICV leptin administrations for multiple successive days (10 µg for four [18] or five [19] successive days) or leptin’s effects on food intake when applied in third ventricle (3.5 µg [20], [21]). As was shown by Smagin *et al* [22] a five time higher dose as used for third ventricle injection will yield similar effects for lateral ventricle injections. Therefore our dose of 15 µg is in the same range as previous studies.

**Figure 3:**
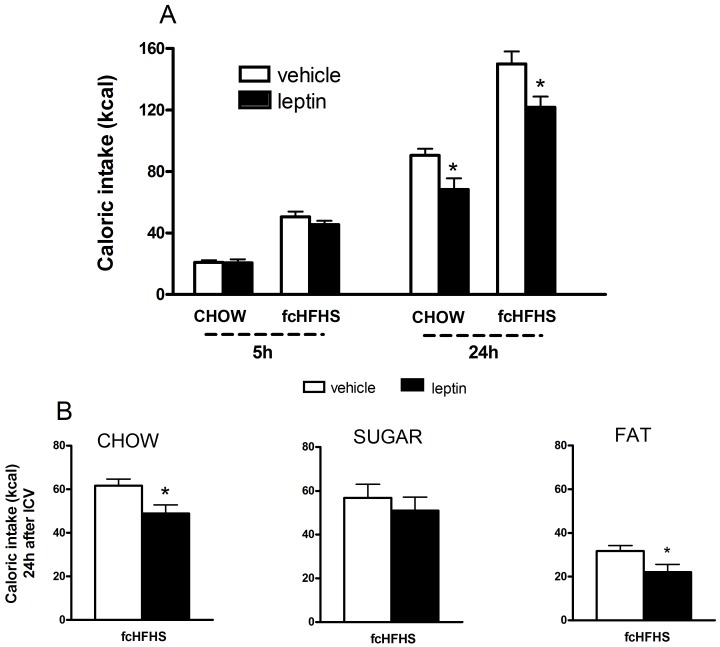
Effect of leptin ICV on caloric intake in rats on a CHOW and a fcHFHS diet. A) ICV leptin suppressed feeding in rats on a CHOW diet and in rats on a fcHFHS diet 24 hours after ICV leptin administration. B) In the fcHFHS group leptin suppressed intake of the chow and fat component, but not sugar. Data are mean ± SEM; *P<0.05, leptin vs. vehicle.

After 8 days diet exposure to fcHFHS or CHOW diet, the effect of ICV leptin or vehicle on caloric intake after 5 h and 24 h was determined. A two-way repeated-measures ANOVA showed an effect of *Time* (F(1,25) = 470.4; p<0.01, *Diet* (F(1,25) = 75.0; P<0.001, and *Leptin* (F(1,25) = 8.3; P = 0.008, and an interaction of Diet*Time: (F(1,25) = 15.9; P = 0.001 and Leptin*Time: (F(1,25) = 9.1; P = 0.006. No interaction was found for the effect of Diet*Leptin: (F(1,25) = 0.22; P = 0.65, or Leptin*Diet*Time: (F(1,25) = 0.14; P = 0.71. Comparisons within single time point groups showed no effect of leptin on 5 h food intake in CHOW or fcHFHS diet, but leptin did suppress feeding in both CHOW and fcHFHS diet after 24 h (P<0.05, Figure 3A). The degree of inhibition was comparable between rats on the CHOW and fcHFHS diet (−23.1±8.1% vs −19.7±4.5%, P = 0.68).

As leptin reduced total caloric intake, the effects on the individual food components were calculated (Figure 3B). The anorectic response of leptin was mainly due to the suppression of fat (P<0.05) and chow intake (P<0.05). Sucrose intake was not significantly affected by leptin. Body weight gain was also reduced 24 hours after ICV leptin (F(1,25) = 4.43; P = 0.046 (data not shown).

### Effect of Leptin on Hypothalamic Expression Levels in fcHFHS vs CHOW Diet

**Figure 4:**
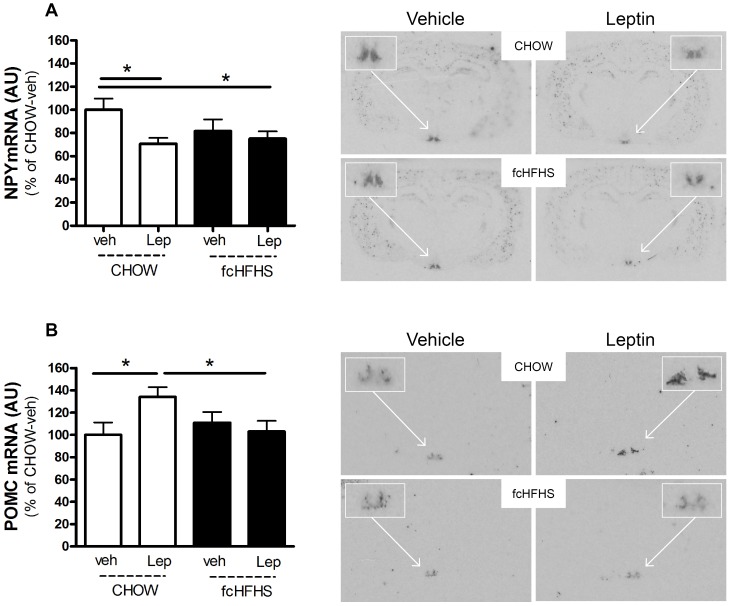
Leptin decreases NPY mRNA and increases POMC mRNA in rats on a CHOW diet, but not in rats on a fcHFHS diet. A) NPY and B) POMC gene expression levels and representative images in the arcuate nucleus of the hypothalamus after ICV dose of vehicle or leptin in rats fed a CHOW or fcHFHS diet. Two-way ANOVA (Diet * Leptin) for NPY P = 0.035, for POMC P = 0.032. Box represents magnification of the arcuate nucleus gene NPY (a) or POMC (b). Values are means ± SEM of seven to nine rats per group. *P<0.05.

In rats on the CHOW diet, leptin significantly decreased NPY mRNA levels in the arcuate nucleus of the hypothalamus (−29±5.2%; P<0.05). In rats of the fcHFHS diet, leptin did not decrease NPY mRNA levels (Figure 4A). Basal NPY mRNA levels were not different between vehicle injected rats on the fcHFHS diet and vehicle injected rats on the CHOW diet.

Basal POMC mRNA levels were not different between vehicle injected rats on the fcHFHS diet and vehicle injected rats on the CHOW diet. Whereas leptin increased POMC mRNA levels in rats on the CHOW diet (34±7.8%; P<0.05), leptin did not change POMC mRNA in rats on the fcHFHS diet (Figure 4B). We found a slight, but non-significant, increase in plasma levels after leptin ICV (data not shown).

### Effect of Leptin on Mesolimbic Expression Levels in fcHFHS vs CHOW Diet

**Figure 5:**
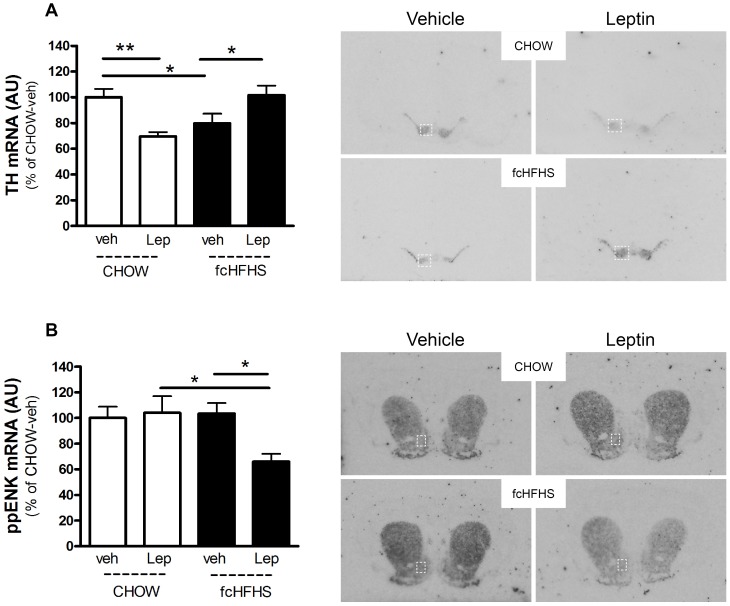
Leptin differentially regulates TH and ppENK mRNA levels in rats on a CHOW and fcHFHS diet. Gene expression levels and representative images for A) tyrosine hydroxylase (TH) mRNA in VTA and B) preproenkephalin (ppENK) mRNA in nucleus accumbens (indicated by box) after ICV dose of vehicle or leptin in rats fed a CHOW or fcHFHS diet. Two-way ANOVA (Diet * Leptin) for TH in VTA P = 0.001; for ppENK in nucleus accumbens P = 0.038. *P<0.05, **P<0.01.

Tyrosine hydroxylase (TH) mRNA was measured in the VTA and substantia nigra. In the VTA, leptin had a differential effect on TH mRNA expression in rats on the CHOW and fcHFHS diet. In rats on the CHOW diet leptin decreased TH mRNA (−30±3.3%; P<0.01). In rats on the fcHFHS diet leptin increased TH mRNA (22±3.1%; P<0.05, Figure 5A). Without leptin treatment, TH mRNA in fcHFHS was significantly lower compared to TH mRNA in rats on CHOW. (CHOW-veh vs fcHFHS-veh: −21±3.0%, P<0.05). In the substantia nigra (pars compacta) a similar pattern was observed for TH mRNA, Two-way ANOVA (Diet * Leptin) P = 0.003.

Preproenkephalin (ppENK) mRNA was measured in the nucleus accumbens and dorsal striatum. ppENK mRNA was not affected by leptin in rats on the CHOW diet, but was significantly decreased by leptin in rats on the fcHFHS diet (−37±7.8% P<0.05, Figure 5B). In the dorsal striatum, a trend towards a similar pattern was observed for ppENK mRNA, Two-way ANOVA (Diet * Leptin) P = 0.06.

## Discussion

Although it has been described that rats on a fcHFHS diet do not respond to peripheral leptin after 1 week diet [3], we here show that, at the same point in time, fcHFHS animals do respond to central administration of leptin in a similar fashion as rats on a CHOW diet. In fcHFHS, leptin decreased the chow and fat component and did not affect sucrose intake. Whereas leptin increased POMC and decreased NPY expression in the arcuate nucleus in rats on a CHOW diet, leptin did not exert an effect on these neural populations in rats on the fcHFHS diet. By contrast, in rats on the fcHFHS diet, leptin decreased nucleus accumbens ppENK mRNA and increased VTA TH mRNA; changes that could explain the effect of leptin on chow and fat intake in rats on a fcHFHS diet when administered centrally.

We show that central leptin decreases caloric intake in rats on a fcHFHS diet, whereas peripheral leptin does not, which supports other studies [23], [24] and suggests that despite peripheral and arcuate nucleus leptin resistance, other areas in the brain are still leptin responsive. Interestingly, we also show that central leptin decreases both chow and fat intake, but not the sugar solution. This agrees with previous data showing suppression of protein and fat intake, but not carbohydrates, by leptin administration in the third ventricle [25]. The finding that leptin in rats on a fcHFHS diet did not affect arcuate POMC and NPY mRNA expression is in correspondence with studies by Enriori et al. [26] and Munzberg et al. [27] that showed leptin resistance of NPY and AgRP neurons in diet-induced obese mice after peripherally administered leptin. It could well be that the increased peripherally circulating leptin levels in rats on a fcHFHS diet has affected the response of these arcuate nucleus neurons to leptin. Indeed, we previously found increased NPY and decreased POMC mRNA levels in rats on the fcHFHS diet accompanied by increased plasma leptin concentrations [16]. This increased NPY and decreased POMC mRNA in rats on a fcHFHS diet was not observed in this study, however, in this study rats were food restricted overnight probably masking effects on NPY and POMC mRNA as these levels respond to fasting [28].

As mRNA expression in the arcuate nucleus was not affected by leptin in rats on a fcHFHS diet, we next determined whether leptin affected other brain areas. Consequently, we focused on the mesolimbic brain area VTA, since dopamine is involved in rewarding aspects of food intake and leptin receptors are localized on dopaminergic neurons [7]–[9]. Therefore, we measured VTA expression levels of tyrosine hydroxylase (TH), the rate-limiting enzyme for dopamine synthesis. Basal TH mRNA levels were downregulated in rats on the fcHFHS diet compared to rats on the CHOW diet (i.e. CHOW-veh). Paralleling our results, most studies report a downregulation in dopamine signaling and decreased TH expression upon high fat diet exposure [29], [30]. The TH mRNA downregulation is likely associated with intake of fat and independent of obesity as dopaminergic changes were observed in animals fed a high-fat diet in the absence of obesity [30]–[32]. The downregulation is not associated with increased leptin levels since *ob/ob* mice (which lack leptin) also show lower VTA TH mRNA levels compared to wild-type mice [13]. ICV leptin in rats on CHOW diet decreased TH mRNA, which corresponds with leptin’s decreasing effects on motivation and downregulation of dopamine signaling described in chow-fed control animals [7], [14], [33], [34].

Interestingly, in rats on the fcHFHS diet, ICV leptin increased VTA TH mRNA. Although this is the first study describing the effect of central leptin on TH expression in a diet-induced obesity model, our results coincide with findings of leptin-deficient *ob/ob* mice and leptin receptor-deficient obese Zucker rats. These models of decreased leptin signaling exhibit decreased VTA and nucleus accumbens TH expression and dopamine content [8], [13], suggesting a positive correlation between leptin and dopamine signaling. Consequently, similar to the leptin-induced increase in VTA TH mRNA in rats on fcHFHS diet, leptin infusion in *ob/ob* mice increases VTA TH protein concentrations [8]. These data suggest that leptin decreases mesolimbic dopamine signaling in (chow-fed) wild-type animals and increases mesolimbic dopamine signaling in leptin-impaired (obesity) models (such as the peripherally leptin resistant fcHFHS rats, leptin-deficient *ob/ob* mice and leptin receptor-deficient obese Zucker rats), pointing to a unique interaction between leptin-function and dopamine signaling, which is intriguing and requires further investigation.

VTA leptin receptor knockdown has been shown to result in increased sensitivity to highly palatable foods [7], [12]. Additionally, wheel running-induced reduction of high fat food preference is linked to increased VTA leptin signaling [35], which supports our findings on the effects of leptin on palatable food (fat) intake decrease. Indeed, although the number of animals was low (N = 7–9 per group) we did find a trend of a negative correlation between fat intake vs. TH expression levels (r^2^ = 0.40, P = 0.12), indicating that the leptin-induced increase of TH mRNA may be correlated to the decrease in fat intake. To what extent TH signaling in fcHFHS rats is directly correlated to changes in palatable food intake remains to be determined in future experiments.

An important cell population in the nucleus accumbens is the dopamine D2 receptor expressing enkephalin neurons. Enkephalins are part of the opioid system and bind to mu opioid receptors (MOR) [36]. Opioids have been strongly implicated in driving palatable food consumption and administration of exogenous MOR ligands in the nucleus accumbens induces hyperphagia of particularly high-fat foods [15]. As the fat component in the fcHFHS diet was significantly decreased upon leptin administration and expression levels of ppENK have been shown to be affected by food anticipation [37] and consumption [38], we next focused on the nucleus accumbens and measured ppENK expression levels. Leptin did not change ppENK levels in rats on the CHOW diet, yet in rats on the fcHFHS diet, leptin significantly decreased ppENK expression levels, which suggests that palatable food items may be necessary for leptin to evoke an effect on ppENK mRNA. A trend towards a positive correlation was found between fat intake vs. ppENK expression levels (r^2^ = 0.49, P = 0.07), suggesting that the leptin-induced decrease in ppENK expression may be correlated to the decrease in fat intake, consistent with the described role of opioids in the nucleus accumbens [15].

Rats on the CHOW diet showed anorectic effects to central leptin, but no differences in ppENK expression, suggesting that the nucleus accumbens was not involved in the anorectic effects in rats on the CHOW diet. Instead, given the significant response of NPY and POMC mRNA upon ICV leptin, the arcuate nucleus has likely contributed to the leptin-induced anorectic effects in rats on the CHOW diet. Considering the lack of involvement of the arcuate nucleus genes in the anorectic effects of ICV leptin in rats on the fcHFHS diet, we speculate that the combined effect of leptin on ppENK and TH mRNA may have at least partly contributed to leptin’s anorectic effects. Our observation of leptin responsive neurons in the mesolimbic system in addition to arcuate nucleus leptin resistance extends work of others that show leptin responsive neurons in brainstem and hypothalamic regions despite arcuate nucleus leptin resistance [26], [27]. We applied leptin in the lateral ventricle to reach a broad range of brain areas. Future studies aimed at local leptin injections may help to determine whether the effects on feeding and gene expression levels upon leptin ICV were due to direct effect of leptin on the mesolimbic brain areas or indirectly via other brain areas such as the lateral hypothalamus [13].

Interestingly, the substantia nigra and dorsal striatum also responded to leptin in fcHFHS rats, which may suggest a role for leptin in regulation of the nigrostriatal pathway [9], and is consistent with the observation that leptin receptors in the substantia nigra mainly project to the dorsal striatum [39]. These leptin effects may relate to regulation of locomotor activity [7], [40] and the motoric effects of food intake regulation, which needs to be further explored in future studies. The anorectic effects by central leptin in the face of arcuate nucleus and peripheral leptin resistance in fcHFHS rats, raises the question whether a longer duration of the fcHFHS diet would also abolish central leptin’s anorectic effects and whether this is paired with progression of neuronal leptin resistance in any of the brain areas, outside the arcuate nucleus, containing leptin receptors [41]. Therefore the effects of chronic fcHFHS exposure on central leptin’s feeding effects and neuronal responsiveness remain to be determined.

In this study the effect of leptin ICV on feeding behavior and leptin ICV on gene expression levels was determined on separate days to prevent confounding effects of food intake on gene expression levels. Future studies may help to determine whether the leptin ICV-induced effects on gene expression levels and feeding behavior are also causally related.

In conclusion, although fcHFHS rats are resistant to peripheral leptin and the arcuate nucleus POMC and NPY neurons are unresponsive, central leptin still reduces food intake in rats on the fcHFHS diet. The VTA and nucleus accumbens are still responsive to leptin in fcHFHS rats and may therefore, at least partly, account for the leptin-induced feeding suppression in rats on a fcHFHS diet.
